# Diagnosis and treatment of hemophagocytic histiocytic sarcoma in a cat

**DOI:** 10.1177/2055116920957196

**Published:** 2020-10-19

**Authors:** Brian Huber, Marc Leleonnec

**Affiliations:** 1 University of Tennessee, Knoxville, TN, USA; 2 Sound Diagnostics, Garden City, NY, USA

**Keywords:** Anemia, hemophagocytosis, histiocytic sarcoma, histiocytosis, neoplasia, splenectomy, splenomegaly, thrombocytopenia, ultrasound

## Abstract

**Case summary:**

A 7-year-old spayed female domestic shorthair cat was presented for persistent anemia of unknown origin. Splenomegaly was diagnosed through physical examination and abdominal radiographs, and an abdominal ultrasound was performed. The ultrasound showed splenomegaly, as well as one discrete mass in the body of the spleen. A splenectomy was performed and histopathology along with immunohistochemistry for CD18 and CD204 confirmed a diagnosis of hemophagocytic histiocytic sarcoma (HS). Two courses of lomustine (CCNU) chemotherapy were used in this cat along with adjuvant oral prednisolone and iron dextran injections, but the cat eventually succumbed to hepatic failure, presumed to be secondary to metastatic disease.

**Relevance and novel information:**

The clinical pathology results and ultrasonographic studies performed before and after treatment in this cat provide useful preliminary information about the biologic behavior and response to treatment for this rare cancer. This is also the first reported case where both CD18 and CD204 were strongly positive, confirming hemophagocytic HS in a cat.

## Introduction

Histiocytic diseases in cats and dogs derive from either dendritic or macrophage lineage.^1^ Dendritic cells (DCs) include interstitial DCs and intraepithelial DCs (Langerhans cells); the majority of canine histiocytic sarcomas (HSs) arise from interstitial DCs.^2^ Macrophages represent the lineage involved with the hemophagocytic syndrome. The origin of the various histiocytic diseases can be determined by cell markers, some of which can be used on formalin-fixed tissue, while others require fresh/frozen tissue, or by flow cytometry.^1^ Information on markers are best described in dogs, where multiple different cell markers have been identified to determine the cell of origin.^2–4^ The macrophage cell lines express beta-integrin markers CD11 and CD18; therefore, tumors arising from macrophage lineages often strongly express CD11d and CD18.^2^ This gives a basis to test for these antigens when confirming the presence of HS. An additional macrophage receptor, CD204, has been shown to be a reliable predictor for histiocytic neoplasia. It is especially useful as it does not react with other round-cell tumors such as lymphosarcoma, or mast cell tumors. CD204 is not expressed with normal DCs, which allows for differentiation with similarly presenting non-neoplastic disease.^1,2^ Canine histiocytic diseases can be divided into malignant and non-malignant forms, with the malignant forms being more common. The most common malignant variant is the HS complex frequently seen in the Bernese Mountain Dog, which has been shown to have a genetic etiology in some cases. The inheritance trait has been isolated to a tumor suppressor gene loci of *CDKN2A/B, RB1* and *PTEN*.^3^ Occasionally, dogs with HS develop severe anemia, which may be accompanied by thrombocytopenia, and this syndrome is referred to as hemophagocytic histiocytic sarcoma (HHS).^4^ Anemia is most likely due to the marked erythrophagocytosis by neoplastic macrophages in the liver, spleen and bone marrow. Thrombocytopenia is likely derived from multiple causes, including consumption, as well as phagocytosis by the deranged neoplastic macrophages.^2,5^

Clinical findings and ultrasonographic interpretation of HS in cats are very limited in veterinary publications, and reports describing the hemophagocytic form of the disease are rare.^6^ This case study describes the diagnostic test results, clinical course of disease and response to therapy in a cat with HHS. To our knowledge, this is only one of four published reports including cats with presumed HHS, and the first with serial hematology and ultrasonography findings after treatment. Presumed HHS is characterized clinically with the findings of pale mucous membranes, anemia, thrombocytopenia and hypoproteinemia.^4,6^

The goals of this case report were to highlight clinical findings key to the diagnosis of HHS in cats, and to document the serial changes in hematology and imaging that may be seen after therapy.

## Case description

A 7-year-old spayed female domestic shorthair cat was presented for decreased appetite and lethargy. A complete blood count (CBC) revealed a borderline anemia (hematocrit 28%) and a serum biochemical analysis was normal. No treatment was initiated. The cat was re-evaluated 2 months later, and CBC and serum biochemical profile were repeated. At this time the anemia was slightly worse (hematocrit 26%) and thrombocytopenia was now present (platelet count 80 × 10^9^/l; reference interval [RI] 200–500 × 10^9^/l [see Table 1]). The white blood cell count and serum biochemical profile were both normal. All leukograms and platelet counts were reviewed by Antech pathologists, and each CBC was performed using the same Siemens Advia 120 CBC analyzer for standardization of values.

**Table 1:** Chronologic representation of hematologic trends, as well as information depicting course of treatment and weight decline

Days	RBC values	WBC values	Platelet count (RI 200–500 × 10^9^/l)	Medication prescribed	Chemotherapy	Weight (kg)
0	RBCs 5.9 × 10^12^/l* HCT 26%^†^ HGB 78 g/l^‡^ MCV 44 fl^§^	WBCs 13.6 × 10^9^/l^¶^ NEUs 11.4 × 10^9^/l^∞^	80 × 10^9^/l	None	None	3.68
20	RBCs 5.0 × 10^12^/l HCT 20% HGB 62 g/l MCV 39 fl	WBCs 24.3 × 10^9^/l NEUs 22.6 × 10^9^/l	143 × 10^9^/l	Prednisolone 1 mg/kg PO q24h Iron dextran (50 mg) 0.5 ml IM	None	3.59
49	RBCs 2.86 × 10^12^/l HCT 11% HGB 41 g/l MCV 39.2 fl	WBCs 41.3 × 10^9^/l NEUs 32.9 × 10^9^/l	102 × 10^9^/l	Prednisolone 1 mg/kg PO q24h	7.5 mg CCNU PO	3.27
63	RBCs 3.5 × 10^12^/l HCT 17% HGB 51 g/l MCV 49 fl	WBCs 36.2 × 10^9^/l NEUs 34.4 × 10^9^/l	149 × 10^9^/l	Prednisolone 1 mg/kg PO q24h Iron dextran (50 mg) 0.5ml IM	None	2.93
65	RBCs NA HCT NA HGB NA MCV NA	WBCs NA NEUs NA	NA	Prednisolone 1 mg/kg PO q12h	None	NA
76	RBCs NA HCT 18% HGB NA MCV NA	WBCs NA NEUs NA	NA	Prednisolone 1 mg/kg PO q12h	7.5 mg CCNU PO	2.75
90^#^	RBCs NA HCT 33% (icteric) HGB NA MCV NA	WBCs NA NEUs NA	NA	None	None	3.05 (ascites)

* Reference interval (RI) 5.0–10.0 × 10^12^/l

† RI 28–45%

‡ RI 9.8–15.4 g/l

§ RI 39–55 fl

¶ RI 5.5–19.5 × 10^9^/l

∞ RI 2.5–12.5 × 10^9^/l

# Day 90 represents data presented on emergency shortly prior to euthanasia. Only an in-house hematocrit was obtained, and serum was icteric grossly

RBC = red blood cell; WBC = white blood cell; HCT = hematocrit; HGB = hemoglobin; MCV = mean cell volume; NEU = neutrophil; NA = not available; CCNU = lomustine

Thoracic and abdominal radiographs, along with an abdominal ultrasound, were performed owing to the worsening CBC trend. Thoracic radiographs showed no alterations within the pleural cavity, and no signs of metastatic disease or primary lung masses. The heart and lungs appeared normal on both the lateral and ventral dorsal projections. There was an opacity visualized silhouetting with the spleen on the right lateral projection. This opacity was anywhere from 1.5 cm to 3 cm in length in other projections, and was diagnosed as splenomegaly with a mass effect present.

**Figure 1:**
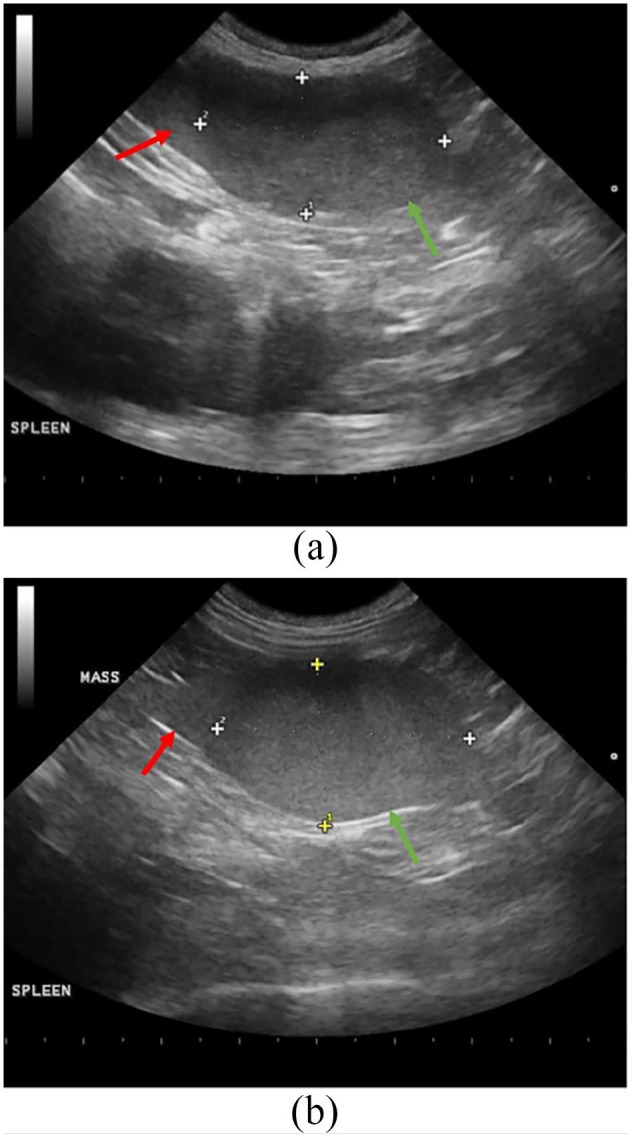
(a) Ultrasonographic image of the spleen on sagittal plane view at presentation. The normal splenic capsule (red arrow) can be seen on the left and transitioning to the capsule-disrupting mixed echogenic mass (green arrow) on the right measuring 3.15 cm × 1.76 cm. (b) Ultrasonographic image of the spleen on transverse plane view at presentation. The normal splenic capsule (red arrow) can be seen on the left and transitioning to the capsule-disrupting mixed echogenic mass (green arrow) on the right measuring 3.25 cm × 2.13 cm

The abdominal ultrasound (Figure 1) showed diffuse splenomegaly with splenic thickness exceeding 1.5 cm, and with one discrete mixed echogenic mass located within the parenchyma of the spleen. The mass was vascular, as evidenced by color Doppler, with very defined margins measuring 3.15 cm and 1.76 cm in a sagittal plane (Figure 1a), and 3.25 cm by 2.13 cm in a transverse plane (Figure 1b). No lymphadenopathy or evidence of metastasis were seen, and the liver lobes were unremarkable with appropriate echogenicity. Based on the appearance of the spleen on ultrasound, this was presumed to be a neoplastic process.

**Figure 2:**
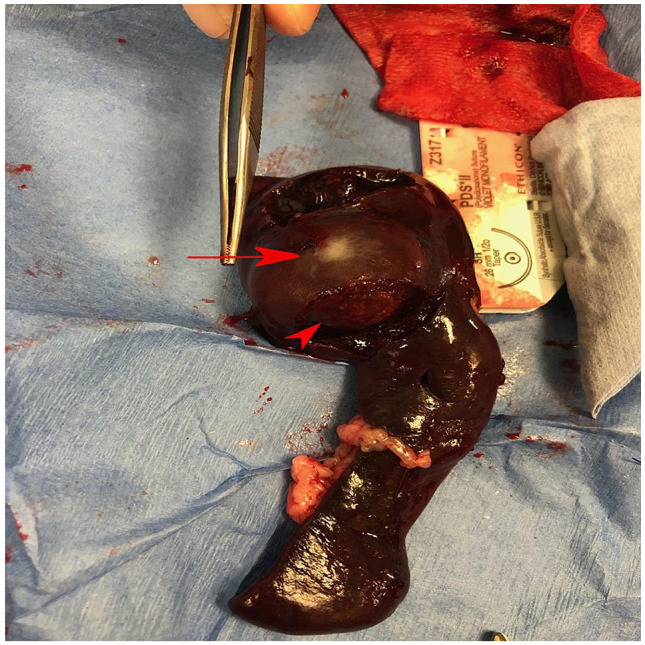
Gross image of the spleen immediately after removal. The mass (red arrowhead) can be seen enlarging the dorsal extremity of the spleen. There was a solitary area of stippled white foci (red arrow) located at the center of the mass. The overall splenic dimensions grossly were approximated at a length of 15 cm and a maximum width of 7 cm

The top three differential diagnoses for this case were mast cell tumor, lymphosarcoma and hemangiosarcoma; therefore, the decision was made to pursue a splenectomy. During surgery, the liver parenchyma was smooth and homogeneous throughout the lobes, and no biopsy was taken. The spleen appeared enlarged primarily across the dorsal extremity and the color was red to dark red. There were no signs of hemorrhage on or surrounding the spleen. The mass could easily be identified as it was lighter red to tan in color, with an area of white stippled foci in the center of the mass (Figure 2).

**Figure 3:**
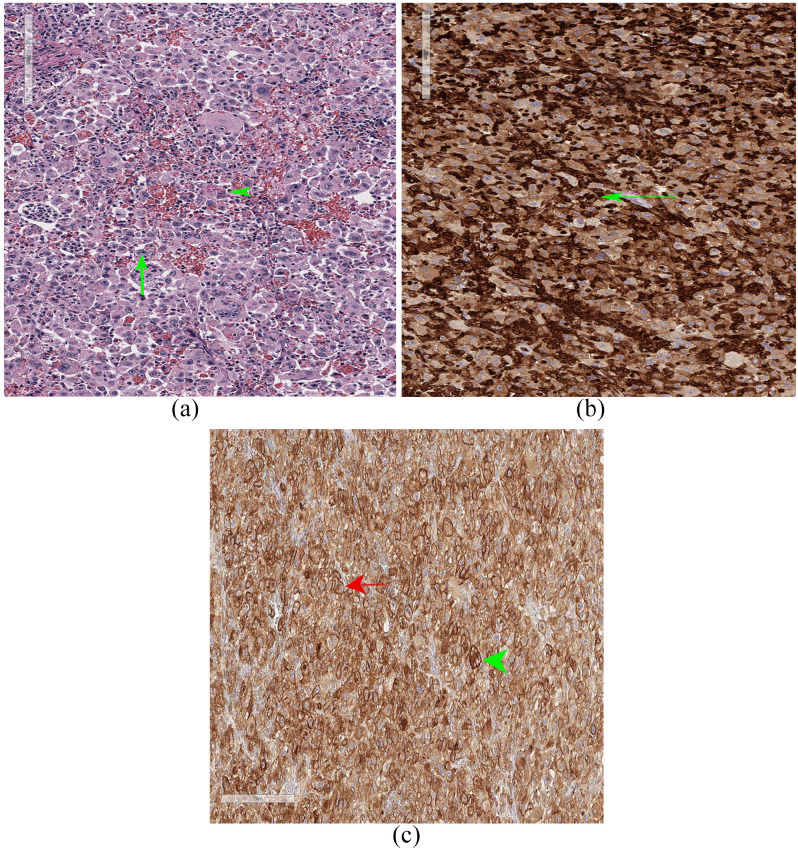
(a) Final hematoxylin and eosin-stained sections of the splenic mass. The majority of the neoplastic cells demonstrate pleomorphism and a sheet-like arrangement with 30 bizarre mitotic figures per high-power field (green arrow). Neoplastic cells are often seen containing phagocytized red blood cells or hemosiderin (green arrowhead). *Courtesy of Set Sokol DVM, DACVP*. (b) Positive immunohistochemistry staining in tumor tissues. Neoplastic cells label positive for CD18 antigen, confirming leukocyte origin and supporting a diagnosis of histiocytic sarcoma (green arrow). *Courtesy of Anna Barthel DVM, DACVP, and Michigan State University Diagnostic Laboratory*. (c) Showing strongly positive staining immunohistochemistry for CD204 in tumor tissues. The neoplastic cells label positive for the CD204 antigen, confirming histiocyte origin (red arrow). There is also hemosiderin present (green arrowhead). *Courtesy of Anna Barthel DVM, DACVP, and Michigan State University Diagnostic Laboratory*

After removal, the spleen was sectioned and submitted for histopathologic evaluation, which yielded a diagnosis of HS, with a high suggestion for the hemophagocytic phenotype (Figure 3a). There was an infiltrating and expanding poorly demarcated neoplasm identified, composed mainly of pleomorphic neoplastic round cells arranged in sheets. The neoplastic cells seen had distinct borders with abundant eosinophilic cytoplasm. Anisocytosis and anisokaryosis were seen, along with multiple binucleate cells. The overall mitotic count was 30 per 10 high-power fields, with a large proportion of these being bizarre in appearance. Neoplastic cells were seen with phagocytized red blood cells and hemosiderin pigment. A Giemsa stain was performed and was negative, ruling out mast cell neoplasia. A diagnosis of HS was confirmed with CD18 immunohistochemistry previously validated for use in feline tissues. The neoplastic cells labeled positive for CD18 antigen (Figure 3b), consistent with a diagnosis of HS (Michigan State University Diagnostic Laboratory). Immunohistochemistry for CD204 was also performed and was strongly positive, further confirming the histiocytic origin of this lesion (Figure 3c; Michigan State University Diagnostic Laboratory).

**Figure 4:**
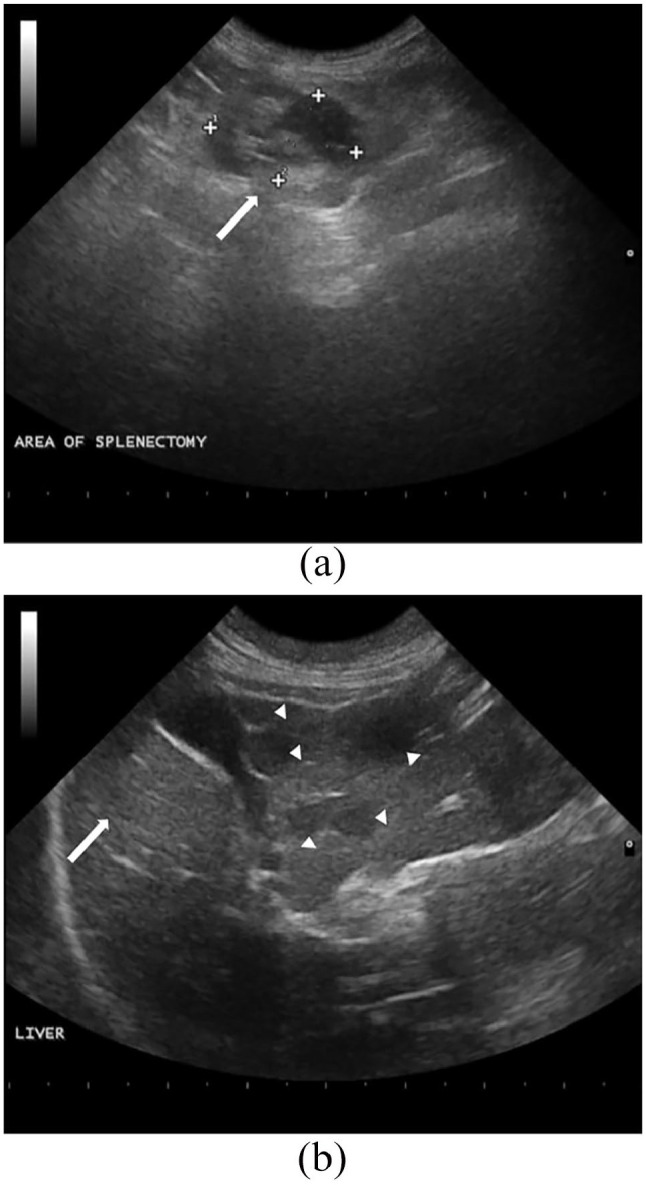
(a) Ultrasonographic image showing a lesion in the area of the previous splenectomy (white arrow), which has a mixed echogenic appearance that measures 1.86 cm × 1.18 cm. (b) Ultrasonographic image of the liver parenchyma highlighting several hypoechoic nodules (white arrowheads) measuring between 0.5 cm and 1 cm in diameter. There were also some unaffected liver lobes (white arrow). The liver on the previous ultrasound showed no evidence of nodules nor alterations in the shape, size or echotexture

Upon consultation with a medical oncologist, chemotherapy with CCNU (lomustine; Wedgewood Pharmacy) was advised in conjunction with oral prednisolone and iron dextran injections. Table 1 shows the timeline of rechecks, medications prescribed, chemotherapy given and pertinent laboratory values. A bone marrow aspirate was discussed and would have assisted with tumor staging and prognostication but was declined by the owner. A recheck abdominal ultrasound performed 58 days postoperatively and after one dose of CCNU showed a mass in the area of the splenectomy and numerous variably sized hypoechoic nodules throughout the liver (Figure 4). The liver nodules were interpreted to be metastatic nodules from the previously diagnosed HHS, although confirmatory fine-needle aspiration cytology was not performed. The complex lesion measuring 1.86 cm in the area of the previous splenectomy (see Figure 4b) was highly suspicious for regrowth or metastasis of the HHS, although fine-needle aspiration cytology of this lesion was not performed either. The liver parenchyma was visually normal both during surgery and on the first ultrasound examination.

Chemotherapy was discontinued following the second ultrasound examination. The owner elected humane euthanasia 90 days post-splenectomy because of progressive anemia and declining clinical condition, including marked peritoneal effusion and jaundice. The owner declined further laboratory analyses and necropsy examination.

The hematologic trends seen (Table 1) in this cat represent steady progression of disease with little to no response to treatment. The anemia initially seen was a mild normochromic–normocytic anemia with a mild inflammatory leukogram. There was a noticeable increase in white blood cell count throughout the course of treatment, and the platelet count did improve after the splenectomy was performed shortly after day 0. The progression to a severe anemia occurred rapidly, even in the face of two separate doses of CCNU, iron dextran injections and continued prednisolone administration. A blood transfusion was discussed; however, the cat declined more rapidly than anticipated.

## Discussion

Histiocytic disease is poorly understood in cats when compared with dogs. This is due to an extremely low prevalence and difficult ante-mortem diagnosis.^1,7^ In canine patients, the diagnosis of HHS requires immunohistochemistry for CD11d and CD18, as these surface markers are present on macrophage cell lines.^1,2,5,7^ Feline HHS is also thought to arise in macrophages based on lack of CD1 expression, but an origin from splenic red pulp macrophages could not be confirmed owing to lack of expression of CD11d in cats.^8^ The most well-documented histiocytic syndrome in cats is feline progressive histiocytosis (FPH). This is comprised mainly of a proliferation of Langerhans cells and most frequently causes skin lesions, including crusting lesions and cutaneous nodules/masses of epithelial origin.^7^ FPH has a slower progression than HS, and FPH does not typically involve the spleen, which is why it was not considered as a differential in this case.

Canine HHS often carries a grave prognosis with a reported median survival time (MST) of 49 days.^2^ The characteristics in both cats and dogs include a regenerative or non-regenerative anemia, thrombocytopenia, hyperbilirubinemia and lymphopenia.^5^ It is unclear if an overall leukocytosis is present in all or most cases, but this was distinctly present in this case. This leukocytosis was characterized mainly by a marked neutrophilia. A neutrophilia in this cat could have developed as a result of several different mechanisms such as an inflammatory response, rebound from chemotherapy and use of steroids, or a paraneoplastic response. Severe anemia and thrombocytopenia were noted in this cat throughout the course of treatment, which is consistent with the most life-threatening problems affecting dogs with this disease.^1,2,5^

Given the highly metastatic nature of this disease, chemotherapy is indicated. In dogs, CCNU has been shown to increase the MST to as long as 568 days with the non-hemophagocytic form of HS.^9^ Dogs with HHS have a reported MST of just 49 days.^9^ The survival time of this cat with HHS was substantially longer at 90 days. CCNU is the chemotherapeutic agent of choice in dogs with HHS and has been shown to have efficacy in cats with neoplasia.^9,10^ Furthermore, the hepatotoxicity often seen with CCNU treatment in dogs appears relatively uncommon in cats, based on phase I and phase II clinical trials.^10–12^ CCNU does appear to have been well tolerated by this cat, as neutropenia or worsening thrombocytopenia were not observed.^10–12^ However, even though the 90-day post-splenectomy survival time of this cat is longer than the previously reported MST of 49 days in dogs with HHS, a definitive antitumor response was not actually documented in this case. In addition, while this cat’s terminal decline was felt to be most likely the result of progressive HHS, complicating concurrent problems such as CCNU hepatotoxicity, intra- or extravascular hemolysis, or hepatic lipidosis were not definitively ruled out.

Multiple factors made the clinical response to treatment difficult to assess in this cat. The cat was not completely staged at diagnosis, as a baseline bone marrow aspiration was not performed. Not all diagnostic tests (ie, bloodwork and abdominal ultrasonography) were performed at every recheck. In addition, prednisolone and iron dextran injections were provided as palliative therapy and to provide some anti-inflammatory effects. Prednisolone was chosen based on improved outcomes in cats with other tumors.^13^ However, new studies in dogs with HS are contending prednisone may lead to faster tumor progression.^4,14^ There remains to be definitive links between prednisone or prednisolone use and time to tumor progression in cats, and therefore prednisolone remained in this protocol. It is important to note the anemia persisted throughout this entire course of treatment with little improvement, despite the cytoreductive attempt of the splenectomy. This suggests a lessened response to the CCNU in this cat, as well as progression of disease despite treatment. This finding of decreased or limited response to CCNU has been reported before in one other case of a cat with HHS. Therefore, further trials need to be performed regarding dosage and efficacy for use in cats.^6,15^

The immunohistochemistry performed was essential in confirming the diagnosis of HHS in this cat. The CD18 and CD204 immunohistochemistry confirmed a neoplasm of histiocytic origin and allowed an appropriate chemotherapeutic plan to be implemented. The diagnosis of HHS was further supported by the aggressive hemophagocytic nature of the tumor seen on the initial hematoxylin and eosin preparation, as well as the negative Giemsa stain. It has been shown that in dogs with diagnosed HS, CD204 stains strongly positive, whereas in other round cell tumors such as lymphosarcoma and most mast cell tumors CD204 staining is negative.^16,17^ Historically, the class A macrophage receptor CD204 has been proven to be very reliable when establishing histiocytic neoplasia origin in dogs and other mammals as well.^16,18^

The ultrasonographic findings and CBC trends in this case did provide novel information with regard to HHS in cats. A comparison of ultrasound images of the affected organs before and after treatment is unprecedented in veterinary literature for cats. The size and echogenicity of masses can vary with HHS, and this case study provides a baseline for cats. The rapid metastasis post-splenectomy in this case is consistent with the poor response to chemotherapy seen with HHS in dogs. The histopathologic features here are comparable to those seen in dogs, but further work is needed to confirm that this disease is similar between the two species. This case is also the first where both CD18 and CD204 were performed and were strongly positive in the cat to confirm HHS. The steady weight decline should also be considered a negative prognostic indicator for HHS in cats as this has been correlated with a negative impact on survival in cats with other neoplasia.^19^

## Conclusions

The unusual neoplasm in this cat made it difficult to predict the poor prognosis prior to the splenectomy as HHS in cats is so rare. Abdominal ultrasonography was critical in this case for characterizing the neoplasia and prompting surgery. The follow-up ultrasound emphasized the aggressive nature with this disorder showing progressive changes to the liver and an abnormal lesion in the area of the splenectomy site. Ultrasonography with fine-needle aspiration or CT scanning may have led to an earlier diagnosis in this cat when anemia was less severe. The immunohistochemistry for CD204 is seldom performed in cats, but proved valuable in this case. There is no overwhelming evidence in this case that CCNU had significant effects on suppressing tumor progression or metastasis. Therefore, treatment of future cases may include alternative chemotherapeutic agents such as doxorubicin. Adding additional reports to the literature will help to raise a level of awareness about this disease in cats and help to guide clinicians in decision-making and prognosis in the future.
